# Ongoing human-mediated spread and hybridization of two major invasive termite species

**DOI:** 10.1098/rspb.2025.0413

**Published:** 2025-05-21

**Authors:** Thomas Chouvenc, Ericka E. Helmick, Alvin Brown, Joseph F. Velenovsky, Sang-Bin Lee, Johnalyn M. Gordon, Brian W. Bahder, Nan-Yao Su, Hou-Feng Li

**Affiliations:** ^1^ Fort Lauderdale Research and Education Center, University of Florida Institute of Food and Agricultural Sciences, Davie, FL, USA; ^2^ South Florida State College, Avon Park, FL, USA; ^3^ Entomology and Nematology, University of Florida, Ft Lauderdale, FL, USA; ^4^ University of California Cooperative Extension, Agricultural and Natural, University of California System, Fairfield, CA, USA; ^5^ Department of Entomology, National Chung Hsing University, Taichung, Taiwan; ^6^ Center for Advanced Science and Technology, National Chung Hsing University, Taichung, Taiwan

**Keywords:** termite, biological invasion, hybridization, anthropomorphic dispersal

## Abstract

Human-mediated biological invasions can lead to introgressive hybridization events between lineages that have evolved independently, with potential for evolutionary, ecological, economic and social impacts. This study provides evidence for the ongoing spread and hybridization between two major invasive and destructive termite pest species, *Coptotermes gestroi* and *C. formosanus* in Florida. Heterospecific courtship behaviour between alates (winged reproductive caste) of the two species has led to the establishment of F1 hybrid colonies in the field, which have matured and produced F1 hybrid alates. Laboratory backcross attempts confirmed the possibility for F1 hybrid female alates to establish viable F2 colonies with males of either parental species. With the recent documentation of introgressive hybridization between the two species in Taiwan, the current study confirms its independent occurrence in Florida, demonstrating that both *Coptotermes* species can hybridize in areas where their distributions overlap. In Florida, the proximity of field-established hybrid colonies to the large leisure boat industry implies that Florida populations of *C. gestroi, C. formosanus* and their hybrids will continue to serve as a bridgehead source of propagules for further dispersal beyond their current distribution, with a potential for F1 hybrids to spread outside of Florida.

## 1. Introduction

The global anthropogenic movement of organisms maintains continuous propagule pressure that facilitates the establishment of species beyond their native distribution range [1]. While non-endemic organisms have impacts on ecosystems that span (sometimes subjectively) the positive-negative spectrum [2,3], some invasive species have disproportionately altered ecological functioning within their introduced ranges [4–6]. Such invasive species increasingly impose an economic burden on agricultural systems, environmental conservation efforts, and communities in urban and suburban environments [7,8]. The human-mediated dispersal of insect pests with societal impacts, as exemplified by the cosmopolitan spread of the German cockroach (*Blattella germanica*) [9], the common bed bug (*Cimex lectularius*) [10] and the pharaoh ant (*Monomorium pharaonis*) [11], has forced pest management industries around the world to constantly adapt to an ever-changing pest pressure [12]. Among household pests, invasive eusocial insects (certain ant, wasp and termite species) can be particularly problematic and costly to residents of affected communities, as the large insect societies of eusocial pest species can lead to difficulties in managing populations at the colony level and across households [13–15].

Another by-product of biological invasions is the increased opportunity for introgressive hybridization events [16], which can have both ecological implications and long-term evolutionary consequences, with a potential for enhancing invasiveness capabilities [17,18]. There is mounting evidence that the ongoing anthropogenic movement of organisms is increasingly resulting in human-induced hybridization events [19–23]. Such concerns hold true for some eusocial insects [24–26], with rare, documented cases of hybridization in termites [27–29], including species with major pest status [30].

**Figure 1.**
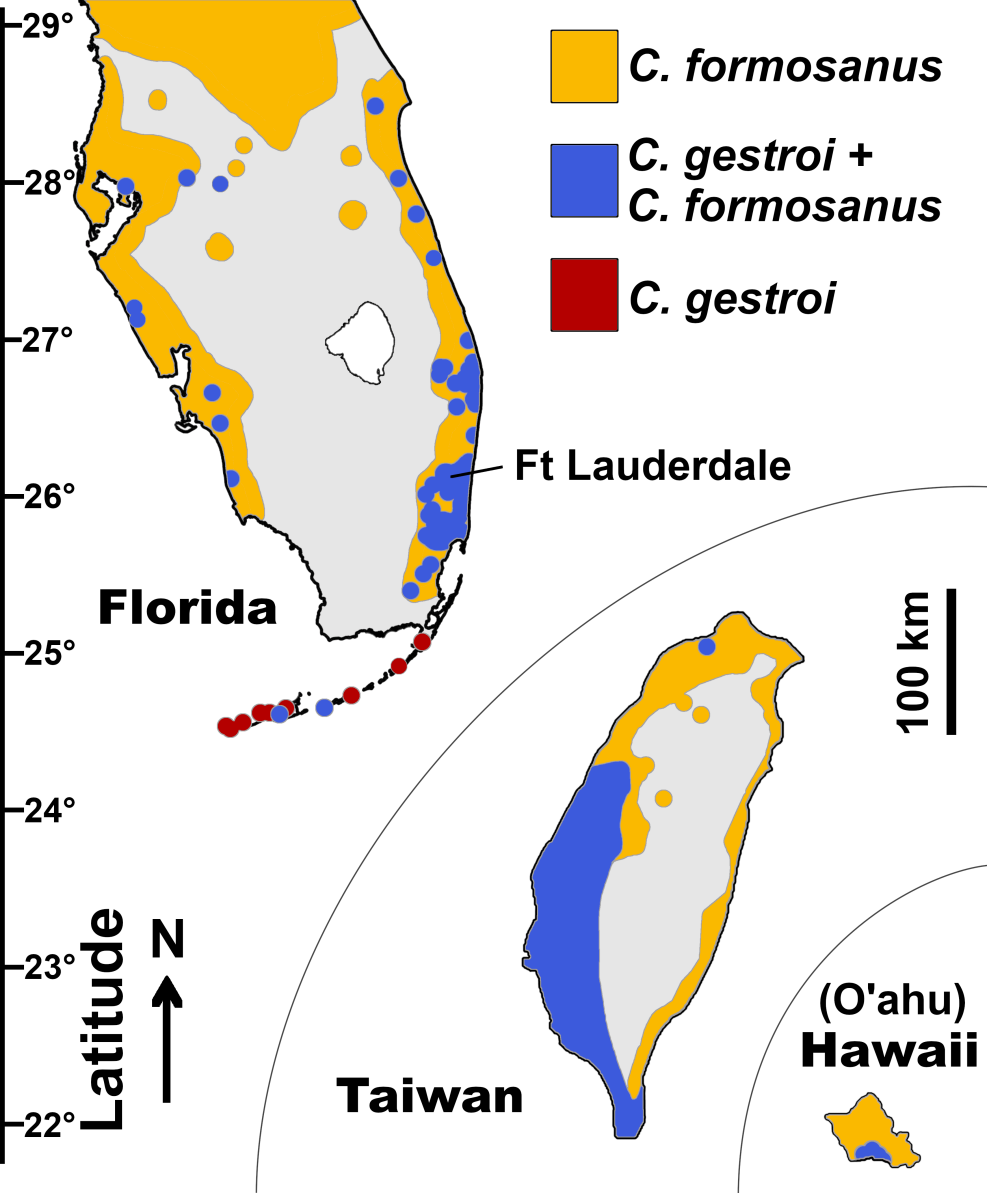
Estimated distribution of *Coptotermes* spp. in areas with documented distribution overlap of *C. gestroi* and *C. formosanus*, as of July 2024. Modified from [45,48,49] and according to updated records from the University of Florida Termite Collection. Scale indicates latitude.

Termites comprise more than 3000 species and are often described as ecosystem engineers owing to their ability to contribute to the decomposition of plant material and bioturbation, especially in the tropics [31,32]. However, a handful of termite genera contain species with status as structural pests, which often feed on timber and commercial wood products, causing significant damage to properties [14]. In addition, the few termite species that display invasive abilities [33] are responsible for most of the estimated $40 billion in termite damage globally and annually as of 2010 [14]. More than half of this global economic impact is attributable to *Coptotermes formosanus* Shiraki (the Formosan subterranean termite) and *C. gestroi* (Wasmann) (the Asian subterranean termite) [34]. These *Coptotermes* species are considered to be two of the most destructive and invasive termite species in the world [35–37]. Both species have continued to expand their distribution ranges within the past century through transoceanic dispersal mediated by maritime vessels, primarily privately owned leisure boats [38–41], while also spreading further inland through the movement of infested material [42]. These two species have distinct climatic requirements [43], with *C. formosanus* now established in subtropical/warm temperate climates in the Northern Hemisphere and *C. gestroi* on its way to achieving a distribution across pantropical climates [44]. These species have evolved in allopatry for 16−18 million years [39], but their distribution ranges now overlap narrowly in three reported locations: southwest Taiwan, south Florida and in the southern part of O'ahu in Hawaii [45–47] (figure 1).

Termite colonies are under the reproductive output of a queen and king, which produce sterile helper castes year-round (workers and soldiers). Once matured, colonies seasonally produce fertile, winged individuals (alates) that engage in dispersal flights to find a partner and initiate a new colony [50]. As of 2010, annual records indicate that *C. gestroi* and *C. formosanus* alates recurrently engage in simultaneous dispersal flight events in southeast Florida [51]. During these events, heterospecific courtship behaviours have been observed in the field [30,52] as female alates of both species share the same sex pheromone [53]. Heterospecific tandem pairs were brought into the laboratory and F1 hybrid colonies were obtained [30]. F1 hybrid *Coptotermes* colonies reared in the laboratory grow similarly to colonies of their parental species [54], have thermal limits and preferences that encompass the climatic ranges of their parental species [55,56], have cuticular hydrocarbon profiles that are intermediate to those of their parental species [57] and have soldiers that display morphotypes that partially depart from either parental species [58].

In the absence of observation of the maturation (production of alates) of hybrid laboratory colonies to date, it has remained unclear if hybrid *Coptotermes* colonies can establish, reach maturity and produce fertile F1 alates in field conditions. It was suggested that, if a potential introgressive hybridization were to occur between the two termite species, it would most likely be first detectable in Taiwan [48], where *C. formosanus* is native and *C. gestroi* is invasive and has established throughout the southwestern part of the island over the past century [59]. Recent field surveys in the city of Taichung, Taiwan [60], confirmed the presence of simultaneous dispersal flight events and *Coptotermes* alates with atypical morphologies were collected, which have been confirmed to be F1 hybrid individuals from both mating combinations (♀ *C. formosanus* × ♂ *C. gestroi* = ‘Hybrid F’, and ♀ *C. gestroi* × ♂ *C. formosanus* = ‘Hybrid G’). In addition, some hybrid alates beyond F1 generations were also collected (at least F2), confirming hybrid introgression in the field in Taiwan, despite no F2 colony viability from preliminary laboratory backcrossing attempts [60].

In comparison, the distribution overlap of *Coptotermes* populations in Hawaii and Florida resulted from more recent human-mediated introduction events. In metropolitan southeastern Florida, *C. formosanus* was first detected in 1980, *C. gestroi* in 1996 [61] and simultaneous dispersal flights were first recorded in 2010 [51]. While the status of putative hybrid *Coptotermes* in Hawaii remains to be investigated further, the ongoing human-mediated spread of both *Coptotermes* species in Florida has resulted in a rapid increase in the overlap of their geographical ranges, now in at least 14 independently introduced counties across coastal cities [45] (figure 1). While the occurrence of simultaneous dispersal flights implies that hybrid *Coptotermes* colonies could establish in the field in Florida, to date, surveys during seasonal dispersal fights have failed to detect hybrid alates [51], initially raising doubts about the assumed viability of field F1 hybrid *Coptotermes* colonies in Florida, until the evidence provided herein.

The current study provides a major update concerning the status of hybrid *Coptotermes* in Florida within the broad context of termite invasion events in the southern US. Alates dispersing from field-established *Coptotermes* colonies were collected in Florida in 2021−2024 surveys. Some collected alates with atypical morphologies were genetically confirmed to be hybrids. F1 hybrid alate fertility and F2 hybrid colony viability were also evaluated through laboratory backcross experiments and, while limited, some F2 hybrid viability was obtained. Finally, the first record of a field-established F1 hybrid colony was confirmed in Florida in 2024. This supports independent ongoing introgressive hybridization of *Coptotermes* in both Florida and in Taiwan. However, in contrast to the Taiwan hybridization case, the Florida *Coptotermes* hybrid population has a high probability to spread outside of Florida, as the field-established hybrid colony was discovered in proximity to a marina, in a city that self-identifies as the ‘yachting capital of the world’. There is, therefore, an exacerbated potential for the invasiveness and destructive potential of these two major invasive termite pest species and their hybrids, far beyond their current distribution range, via sustained human-mediated dispersal.

## 2. Material and methods

### (a) Survey of *Coptotermes* dispersal flights

Between 2014 and 2017, a daily *Coptotermes* dispersal flight monitoring programme was initiated, following an established light trap protocol during sunsets between 15 February and 15 June of each year at a single location in residential Fort Lauderdale (Broward County, FL, USA) [51]. For each dispersal flight event, the daily average alate weight was recorded and alate species and sex were determined from morphological characteristics to obtain a daily census, which served as a proxy to estimate the relative flight activity in the area over time. After 2017, alate monitoring transitioned to casual collection (no metrics collected) to maintain the annual establishment of laboratory colonies from field-collected alates for all four *Coptotermes* mating combinations [54,62]. However, in April 2021, several alates with atypical morphology were collected during such casual alate collection events. As a result, the full seasonal monitoring, including metrics acquisition, was later resumed in 2022, 2023 and 2024, and flight records are reported herein for *C. gestroi*, *C. formosanus* and atypical *Coptotermes* alates (= F1 hybrids) for these three consecutive years between 15 February and 15 June. Results of alate collections are displayed herein as the average daily flight activity (number of alates) over the 3 years. Average alate weights were obtained from subsamples (30 alates or all available alates if less than 30) of independent dispersal events from the 2022 dispersal season (*n* = 24 for *C. gestroi*, *n* = 15 for *C. formosanus* and *n* = 9 for F1 hybrid) and compared with an ANOVA followed by a Tukey Honestly Significant Difference (HSD) post hoc test. An insufficient number of events with F1 male hybrids prevented a reliable estimation of their body weight. When applicable, results are presented as mean ± s.d.

### (b) F2 backcross pairings in the laboratory

Field-collected F1 hybrid *Coptotermes* alates were assessed for their viability and fertility by backcrossing them with either *C. gestroi* or *C. formosanus* alates from the 2021 to 2022 collection events. The colony foundation process and rearing conditions were identical to those previously detailed [62]. All female-male species permutations were established (within the limit of initial biological material availability), including the two parental species (first named individual is always the female in the mating permutations): 40 *C*. *gestroi* (♀ *C. gestroi* × ♂ *C. gestroi*) and 40 *C*. *formosanus* (♀ *C. formosanus* × ♂ *C. formosanus*); the two F1 hybrids: 40 ‘F1 Hybrid G’ (♀ *C. gestroi* × ♂ *C. formosanus*) and 40 ‘F1 Hybrid F’ (♀ *C. formosanus* × ♂ *C. gestroi*); and two backcross mating combinations: 30 ‘F2 Hybrid HF’ (HY×CF = ♀ Hybrid × ♂ *C. formosanus*) and 30 ‘F2 Hybrid HG’ (HY×CG = ♀ Hybrid × ♂ *C. gestroi*). Half of the colonies for each combination were established during the 2021 season, and the remaining half in 2022. A small number of F1 hybrid male alates (*n* = 37) were collected in 2022, and establishment of all three remaining backcross mating permutations were attempted (7 ♀ CG × ♂ HY, 7 ♀ CF × ♂ HY, 7 ♀ HY × ♂ HY). At 1 year of colony growth, all colonies were processed for viability and a census of all castes was obtained. Viable colonies (= presence of a queen, king, viable brood, workers and soldiers) were transferred to 1.5 l containers with additional soil, wood and moisture, and colony growth (number of workers used as a proxy) and viability were determined again at 2 years post-foundation. Statistical comparison of colony growth between mating combinations was performed for each year with an ANOVA and a Tukey HSD post hoc test, although mating combinations with low (<25%) or no colony survival were excluded from the analysis. Colony survival rates among mating combinations were compared using Chi-square tests.

### (c) Molecular diagnostics of F1 and F2 *Coptotermes* hybrids

To determine each individual termite’s pedigree, a qPCR/HRM (quantitative Polymerase Chain Reaction / High-Resolution Melting analysis) diagnostic approach was used, using both mitochondrial and nuclear markers previously optimized for this purpose [60,63]. For alates collected from the field, 40 *C*. *gestroi* alates, 47 *C*. *formosanus* alates and 82 atypical female alates collected between March 2021 and June 2023 were used, for a total of 169 alates. For laboratory-established colonies, workers were used to determine their respective colonies’ genetic makeup, with six workers per colony (three colonies of origin per mating type) for both parental and both F1 hybrid mating types (*n* = 18 individuals per mating type). These laboratory-reared colonies provided control genetic profiles for each confirmed mating combination. For F2 hybrid colonies, a variable number of workers were sampled depending on availability (from 8 to 16 workers from 4 colonies for ‘F2 Hybrid HG’, and from 7 to 16 workers from 7 colonies for ‘F2 Hybrid HF’), for a total of 190 individual workers. The detailed qPCR/HRM protocol is available in the electronic supplementary material (ESM1). Allele identification from HRM profiles were converted as discrete variables for STRUCTURE (v. 2.3.4), which was used to determine the probability of assignment of ancestry for each individual, with k = 2 reflecting the two parental species of origin, 10 000 burn-in steps and 1 00 000 data collection steps. The admixture model with correlated allele frequencies was used.

#### (i) Morphological characters of F1 alates and F2 soldiers

Alate morphology was compared between *C. gestroi*, *C. formosanus* and F1 hybrids following the protocol by [60]. In brief, live hybrid alates display an intermediate dorso-abdominal nutty brown hue, which stems from the golden orange of *C. formosanus* and the dark leather brown of *C. gestroi* alates. In addition, the head capsule colour of hybrid alates, the shape of the antennal spot, and the contrast between the antennal spot and the head capsule are also intermediate relative to the phenotypes of these traits in the two parental species. Differences in soldier head capsule morphology between parental species and F1 hybrid colonies were determined in [58] using the fontanelle shape, the number of setae beside the fontanelle, the presence/absence of a bulging vortex behind the fontanelle and the striations along the postmental sulcus. Examination of the combination of these four traits allows for differentiation among colonies of parental species and F1 hybrid colonies [58]. In the current study, these four traits were assessed in viable F2 hybrid colonies using at least three soldiers per colony of origin to test if F2 soldiers would primarily display traits from the species it was backcrossed with.

### (d) Detection of a field-established hybrid colony

As part of an ongoing large-scale, multi-year survey towards a population management programme for *Coptotermes* colonies infesting trees in city-owned parks, a total of 1304 trees from eight independent parks were each inspected five times for *Coptotermes* infestation between October 2019 and November 2024. Trees were visually inspected as per [49] for evidence of termite activity, and termite samples were systematically collected in 85% ethanol. Soldier morphology was then used for species identification as per [58]. In the current study, we report the detection of *Coptotermes* infesting trees in one of these parks (GPS coordinates: 26.0995−80.1650), revealing conclusive evidence of the presence of an F1 hybrid colony in the field. Identity of soldiers with an F1 hybrid morphology was confirmed using the qPCR/HRM diagnostic approach described herein.

## 3. Results

### (a) *Coptotermes* dispersal flight events and collection of F1 hybrid alates in the field

**Figure 2.**
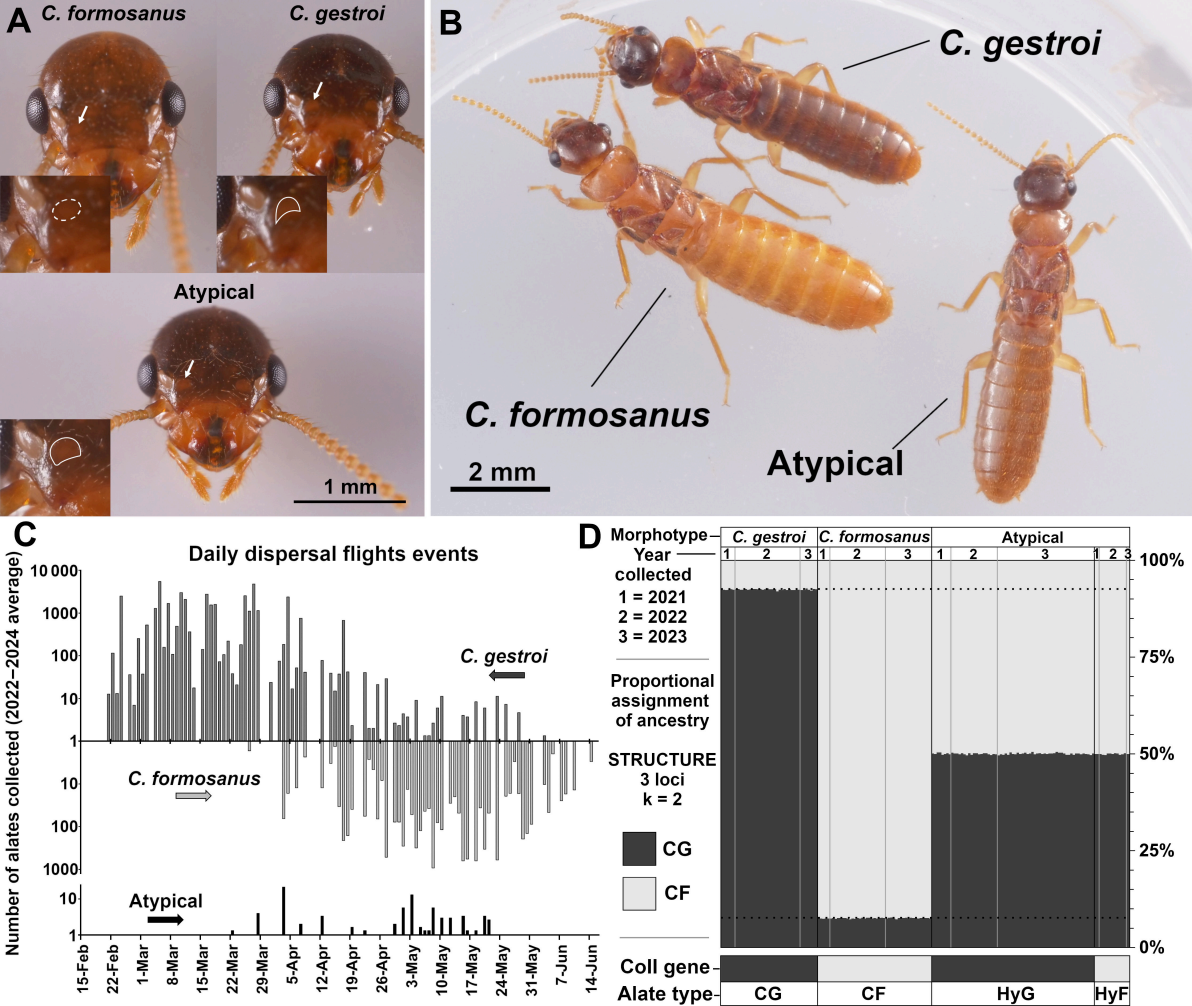
*Coptotermes* alate morphology, phenology and genotype. (A) Focus on the antennal spot morphology (arrows point to respective inset images at scale 2×). The *C. formosanus* spot is poorly defined. There is a defined crescent antennal spot for *C. gestroi,* and there is an enlarged antennal spot for atypical alates. (B) Intermediate colour hue in the atypical alate dorsal abdomen (live individuals) compared with parental species, here all females. (C) Dispersal flight phenology (daily average number of collected alates, on a log+1 scale, over the 2022−2024 seasons) of the three alate morphotypes. (D) Results from a STRUCTURE analysis of microsatellites (169 individuals collected in 2021−2023, k = 2, loci = 3), and their maternal lineage (CoII gene), confirming that atypical alates were produced by field-established Hybrid F and Hybrid G colonies in Florida since at least 2021. The proportional assignment of ancestry from the admixture model is bound to approximately 92.7% certainty for parental species owing to the use of only three loci in the analysis, although sufficient to confirm hybridization. CG = *C. gestroi*, CF = *C. formosanus*, HyG = F1 Hybrid G, HyF = F1 Hybrid F.

In April 2021, while collecting *C. gestroi* and *C. formosanus* alates during simultaneous dispersal flight events for the initial purpose of establishing heterospecific laboratory colonies, 87 *Coptotermes* female alates (but 0 male alates) displaying atypical morphology were obtained from the light trap, along with thousands of *C. gestroi* and *C. formosanus* alates. Morphology of the antennal spots (figure 2A) of the atypical alates resembled the description of F1 hybrid alates collected in Taiwan, with a high contrast between head capsule and antennal spot coloration and antennal spots that were larger than those of *C. gestroi* alates [60]. In addition, live atypical alates displayed an intermediate colour hue of abdominal tergites (figure 2B), and genetic markers confirmed them to be F1 hybrid alates (see below). For female alate body weight, *C. gestroi* alates weighed an average of 7.46 ± 0.41 mg, *C. formosanus* alates weighed an average of 10.30 ± 0.62 mg and F1 hybrid alates weighed an average of 8.15 ± 0.56 mg. These three average weights were significantly different according to an ANOVA followed by a Tukey’s HSD test (F = 144.88, d.f. = 2, *p* < 0.001, and Tukey’s HSD: *p* < 0.01 for all pairwise comparisons). However, the female hybrid alate weight range (7.15–9.01 mg) partially overlapped with that of female *C. gestroi* alates (6.49–8.17 mg) and that of female C. *formosanus* alates (8.73–11.12 mg), preventing the use of body weight as a reliable trait for identification.

The resumption of the alate monitoring programme in 2022−2024 confirmed the annual ongoing presence of hybrid *Coptotermes* alates during dispersal flight events (189 atypical alates collected), including during heterospecific synchronous dispersal flights. Out of the 137 days with recorded *Coptotermes* alate activity over the 3 year period, there were 25 independent days when F1 hybrids were collected (figure 2C) and 94.7% of all collected F1 hybrid alates dispersed at the same time as at least one of the two parental species. When examining the synchronicity of the dispersal flight events over all three seasons for days in which F1 hybrid alates were collected, 52% of these days also had both *C. gestroi* and *C. formosanus* alates, 16% of these days also had *C. gestroi* only, 24% of the days also had *C. formosanus* only and 8% of days had no parental species alates in addition to hybrid alates (see electronic supplementary material, table S2 for additional details). In summary, 26 587 *C*. *gestroi* alates and 7507 *C*. *formosanus* alates were collected, while 37 male and 65 female hybrid alates were collected in 2022. In 2023, 60 391 *C*. *gestroi* alates and 6592 *C*. *formosanus* alates were collected, while 5 male and 35 female hybrid alates were collected. In 2024, 30 210 *C*. *gestroi* alates and 6708 *C*. *formosanus* alates were collected, while 3 male and 44 female hybrid alates were collected. Out of 1 38 182 alates collected over the 2022−2024 period, 84.81% of individuals were *C. gestroi*, 15.06% were *C. formosanus* and 0.13% were F1 hybrids.

A subsample of 82 atypical alates collected between 2021 and 2023 was used for molecular identification confirmation, and all were confirmed to be F1 hybrid *Coptotermes* (figure 2D), as microsatellite markers confirmed heterospecific heterozygosity for *Cg33*, *Clac1* and *Copt10F* alleles. In addition, 81.7% of tested hybrid alates (*n* = 67) were identified as ‘F1 Hybrid G’ (♀ *C. gestroi* × ♂ *C. formosanus*), as they possessed a *C. gestroi* maternal lineage. The remaining 18.3% of hybrid alates (*n* = 15) were confirmed to be ‘F1 Hybrid F’ (♀ *C. formosanus* × ♂ *C. gestroi*), implying that at least two independent field-established hybrid colonies (‘F1 Hybrid G’ and ‘F1 Hybrid F’) were the source of the alates collected during 2021−2023 events when F1 hybrid alates were collected. None of the alates collected displayed allelic profiles beyond the F1 generation. F1 hybrid dealate specimens collected on 13 May 2024, were archived in the University of Florida Termite Collection (UFTC #FL-7567)

### (b) F2 backcross pairings in the laboratory

**Figure 3.**
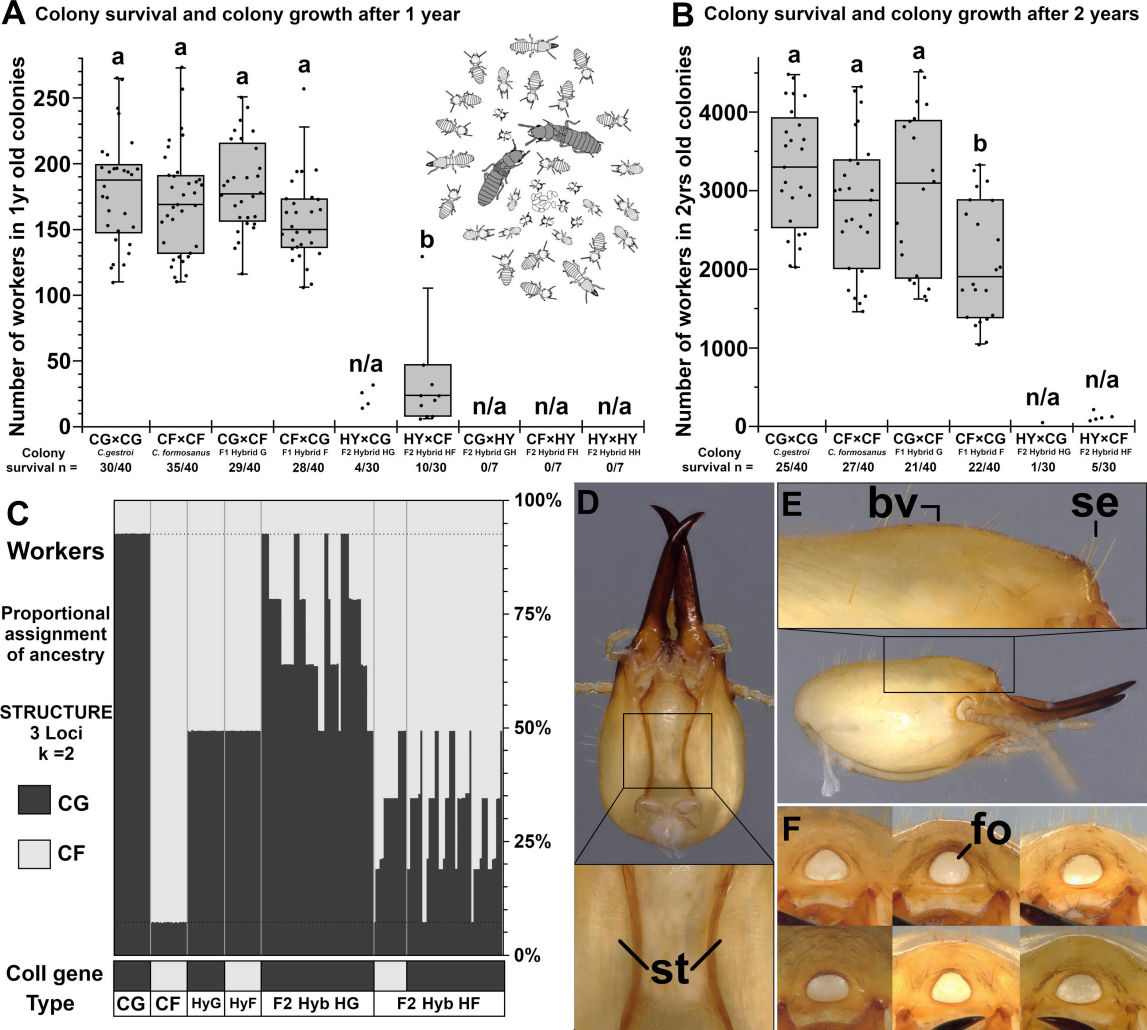
F2 hybrid colonies characteristics. (A) Colony survival and growth 1 year after establishment in laboratory conditions (ANOVA, F = 28.81, d.f. = 4, *p* < 0.001, different letters indicate significant difference in colony growth with a Tukey’s HSD test, n/a = not applicable). (B) Colony survival and growth 2 years after establishment in laboratory conditions (ANOVA, F = 8.07, d.f. = 3, *p* < 0.001, Tukey’s HSD test). (C) Results from a STRUCTURE analysis of microsatellites for 190 workers collected from 23 laboratory colonies ( k = 2, loci = 3), and their maternal lineage (CoII gene), confirming that F1 hybrid female alates can produce viable offspring when backcrossed with either parental species. For F2 workers, individuals from a given colony are ranked in order of ancestry assignment towards the species the hybrid female backcrossed with, to visually determine each colony of origin (4 ‘F2 Hybrid HG’ colonies and 7 ‘F2 Hybrid HF’ colonies). CG = *C. gestroi*, CF = *C. formosanus*, HyG = ‘F1 Hybrid G’, HyF = ‘F1 Hybrid F’, ‘F2 Hyb HG’ = HY × CG and ‘F2 Hyb HF’ = HY × CF. (D) Ventral morphology of F2 soldier head capsule displaying faint striations (*st*) beside the postmental sulcus. (E) Lateral morphology of F2 soldier head capsule displaying a prominent dorsal bulging vertex (*bv*) and two pairs of setae beside the fontanelle (*se*). (*f*) Frontal morphology of six different F2 soldier head capsules displaying highly variable fontanelle shapes (*fo*) that overlap with soldier morphology from both parental species and F1 hybrids.

Laboratory-reared conspecific (parental) and heterospecific (F1 hybrid) colonies (*n* = 40 per mating type) displayed similar colony survival after 1 year (χ^2^ = 4.00, d.f. = 3, *n* = 160, *p* = 0.26, approximately 76% survival) and after 2 years (χ^2^ = 2.36, d.f. = 3, *n* = 160, *p* = 0.51, approximately 64% survival). In addition, they displayed similar colony growth with 173 ± 38 workers produced after 1 year (figure 3A). Colonies from all four mating combinations continued to increase their populations during the second year of colony growth (figure 3B), where ‘F1 Hybrid F’ colonies produced significantly less workers (2101 ± 762 workers) than the three other mating combinations (3029 ± 880 workers in average) (F = 8.07, d.f. = 3, *p* < 0.001).

In comparison to parental species and F1 hybrid colonies, F2 backcross mating combinations mostly resulted in colony establishment failure, with notable exceptions. In colony replicates founded with alates from the 2021 dispersal season, all F2 backcross colonies failed, but some F2 survival and growth were observed in colony replicates founded with alates from the 2022 season. All colonies established from mating combinations using a F1 hybrid male (♀ CG × ♂ HY, ♀ CF × ♂ HY, ♀ HY × ♂ HY backcrosses, *n* = 7 per mating combination) failed to establish, as all F1 hybrid males died within the first few weeks of colony foundation. However, some F2 colonies initiated in 2022 with a F1 hybrid female led to variable establishment success (figure 3A). For ‘F2 Hybrid HG’ (♀ HY × ♂ CG backcross) colonies, 13% (4/30) survived after 1 year, with 23 ± 8 workers produced on average, and a single colony (3%) survived a second year of colony growth, containing 53 workers. In comparison, ‘F2 Hybrid HF’ (♀ HY × ♂ CF backcross) colonies displayed a 33% (10/30) survival in the first year and a 17% (5/30) survival in the second year, which was marginally superior to ‘F2 Hybrid HG’ colony survival (*n* = 60, d.f. = 1, year 1: χ^2^ = 3.35, *p* = 0.067, year 2: χ^2^ = 2.96, *p* = 0.084). After 1 year of colony growth, 9 out of the 10 ‘F2 Hybrid HF’ viable colonies produced 21 ± 13 workers, similar to what was observed with ‘F2 Hybrid HG’ colonies. One ‘F2 Hybrid HF’ colony was an outlier, as it produced 131 workers, which was within the range of what parental and F1 hybrid colonies produced. Overall, while 10 of the 30 ‘F2 Hybrid HF’ colonies were viable after 1 year, their growth was limited when compared with parental and F1 hybrid colonies (figure 3A, F = 28.81, d.f. = 4, *p* < 0.001). After 2 years of colony growth, surviving ‘F2 Hybrid HF’ colonies displayed between 79 and 214 workers, which were colony populations at least one order of magnitude smaller than colonies from parental or F1 mating combinations (figure 3B). Finally, the most successful ‘F2 Hybrid HF’ colony (131 workers after 1 year of growth and 214 workers after 2 years of growth) was processed one last time 10 months later (2 years and 10 months after foundation) and displayed 1037 workers, confirming eventual growth in one of the F2 hybrid laboratory colonies.

For laboratory-reared colonies, individual workers sampled from both parental species and both F1 hybrid mating combinations displayed the expected allelic ancestry assignment (figure 3C). In addition, backcrossed mating combinations resulted in individual F2 workers displaying ancestry assignment ranging from approximately 50% to approximately 92.7% towards their backcrossed species. The four ‘F2 Hybrid HG’ colonies, respectively, displayed 81.2%, 73.1%, 75% and 78.1% for an average of 77.3% *C*. *gestroi* allele inheritance (as ordered from left to right on figure 3C). The seven ‘F2 hybrid HF’ colonies, respectively, displayed 68.7%, 68.7%, 72.9%, 66.7%, 66.7%, 79.2% and 73.8%, for an average of 70.6% *C*. *formosanus* allele inheritance (as ordered from left to right on figure 3C). Such results confirm that these colonies are a generation beyond F1, and colony genetic profiles are all within the 0.65 < qi < 0.85 expected for F2 colonies (approximately 75% towards species it was backcrossed with), with the expected allelic diversity of F2 colonies. Finally, 10 out of 11 tested F2 colonies displayed a *C. gestroi* maternal lineage, while a single colony displayed the *C. formosanus* maternal lineage (one of the ‘F2 hybrid HF’ colonies), confirming that, in Florida, collected F1 hybrid female alates were produced by at least two field-established colonies from both mating combinations (Hybrid G and Hybrid F), and that both types have the potential to backcross with either parental species.

Soldier morphologies from ‘F2 Hybrid HG’ and ‘F2 Hybrid HF’ colonies were compared with parental species and F1 hybrid soldier descriptions [58]. F2 soldiers displayed highly variable intra- and inter-colonial morphologies, but with some consistent patterns. First, the sclerotized striation along the postmental sulcus was faint in F2 hybrid soldiers, as observed in both ‘F1 hybrid F’ and ‘F1 Hybrid G’ soldiers, while it is well-defined and sclerotized in *C. gestroi* but absent in *C. formosanus* (figure 3D). Second, all F2 hybrid soldiers displayed a bulging vertex behind the fontanelle, which is absent in *C. formosanus*, but observed in *C. gestroi* and in both F1 hybrid types (figure 3E). Third, the number of pairs of setae beside the fontanelle, while variable owing to high fluctuating asymmetry, was generally observed at two pairs for F2 hybrid soldiers (figure 3E). Similarly, both F1 hybrid and *C. formosanus* soldiers also have two pairs of setae, while only one pair is observed for *C. gestroi* soldiers. Finally, the fontanelle shape is defined as subcircular in *C. gestroi*, as trianguliform in *C. formosanus* and as horizontally compressed ellipsoid in F1 hybrid soldiers. In comparison, the intra-colonial fontanelle shape of F2 hybrid soldiers was highly inconsistent, varying from circular to trianguliform and even ellipsoid phenotypes (figure 3F). Even if all of these traits are combined, it is not possible to discriminate between F1 and F2 soldier morphologies with certainty, but if a soldier displays a bulging vertex behind the fontanelle, two pairs of setae beside the fontanelle and faint striations along the postmental sulcus, it is likely to be a hybrid soldier of at least F1 generation.

### (c) Confirmation of an F1 hybrid colony established in the field

**Figure 4.**
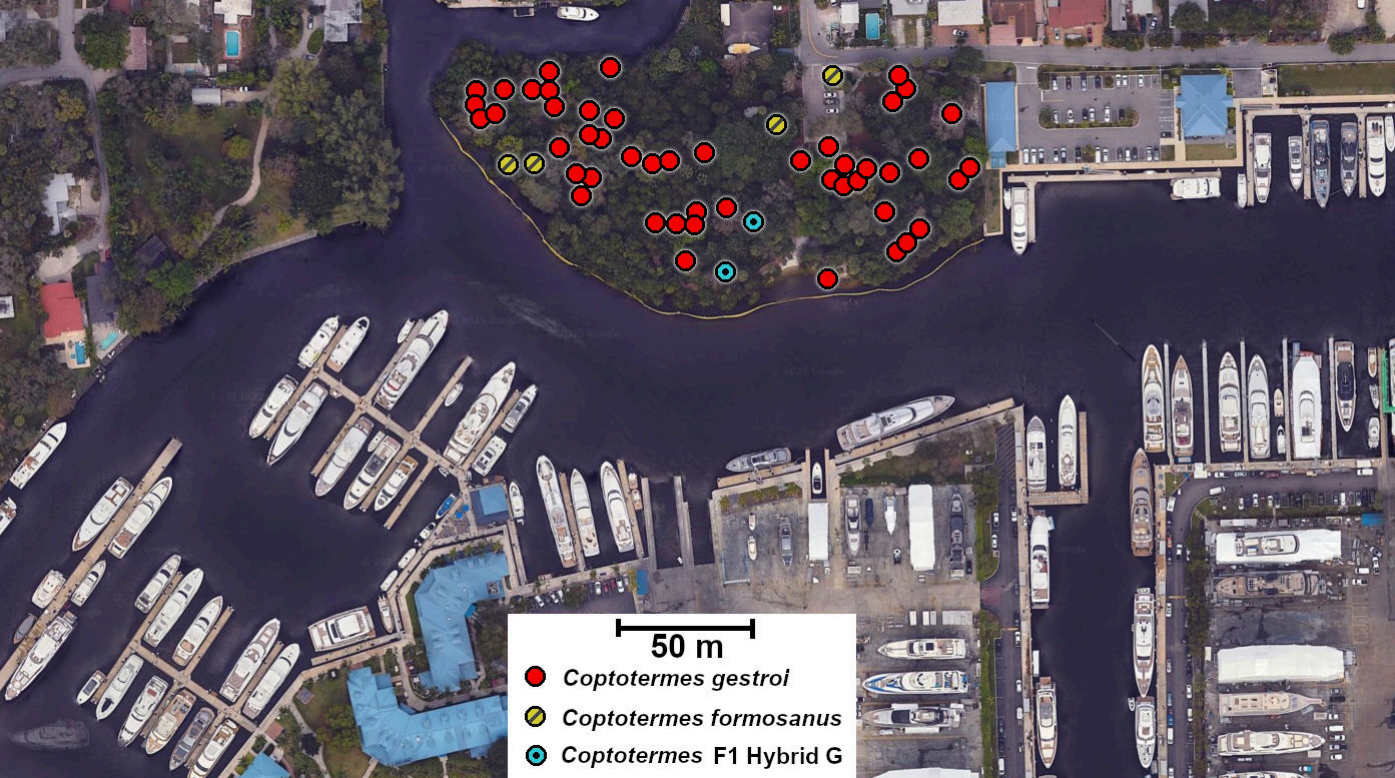
Confirmation of a *Coptotermes* hybrid colony established in the field, in Ft Lauderdale, Florida city. Soldiers collected from two trees located 22 m apart were confirmed to be F1 Hybrid G termites, putatively from the same colony. This park is located within a boat marina where private vessels with maritime capabilities are moored or docked throughout the year. This situation reflects the potential for vessels to be infested by either *Coptotermes* species or their hybrids over time as they come and go, making such a location a functional bridgehead for the further spread of *Coptotermes* in other parts of the world.

As part of an ongoing long-term management pilot project initiated in 2019, trees were visually inspected annually for *Coptotermes* infestation in several Ft Lauderdale, FL city parks. In one of these parks, which contains 326 trees, a total of 52 trees were infested by either *Coptotermes* species between 2019 and 2024. On 30 October 2024, atypical termite soldier samples were collected from two infested trees, which resembled F1 hybrid soldiers as described in [58]. The qPCR/HRM diagnostic confirmed both samples to be ‘F1 Hybrid G’. The two sets of soldier samples were archived in the University of Florida Termite Collection (UFTC #FL-8024 and #FL-8025). The proximity of the two trees (22 m apart) makes it possible for the two collected samples to be from the same colony of origin, but this could not be confirmed with the resolution of the diagnostic. Regardless, this observation represents the first direct evidence of an F1 hybrid *Coptotermes* colony established in the field in Florida (figure 4). In addition, this park is located 1.3 km away from the alate monitoring site used in the current study, making this established F1 hybrid colony an unlikely source for the F1 Hybrid G alates collected in the light trap between 2021 and 2023, confirming that F1 colonies that have the capability to disperse alates have independently established at more than one location. Critically, this park is surrounded by commercial marinas, with hundreds of private leisure watercrafts docked within dispersal flight distances, making boat infestation by F1 hybrid alates a strong possibility.

## 4. Discussion

This study provides evidence of the ongoing spread and hybridization of two invasive *Coptotermes* pest species in at least two distinct locations in the world: Taiwan and Florida. During the 2022−2024 alate monitoring programme in Florida, 0.13% of all collected individuals were identified as F1 hybrids, compared with the ≈4% of hybrid alates from the Taiwan surveys (≈28-fold of the proportion of hybrid individuals from alates collected). This provides support for the hypothesis that the hybridization process in Taiwan has been ongoing for a longer period of time than the more recent hybridization events in Florida [47,60]. The relatively recent establishment of the two termite species in Florida and the existence of the *Coptotermes* alate dispersal flight monitoring programme helped us provide real-time documentation of a pre-hybrid and a post-hybrid era for south Florida, which most likely occurred between 2010 and 2015. After more than a decade of ongoing spread, the first documented simultaneous collection of alates of both species occurred in 2010 [51], the first heterospecific tandem behaviour was observed in 2013 [30] and the first detection of F1 hybrid alates occurred in 2021 (current study). This timeline of events is in line with the requirement that colonies reach maturity > 5 years after foundation before producing alates [50], the idea being that the density of F1 hybrid alates must be high enough to be detectable, and the notion that enough time must pass for some field-established hybrid colonies to be within flying distances of monitoring locations.

In both Taiwan and Florida, both ‘F1 Hybrid F’ and ‘F1 Hybrid G’ alates were collected from the field, confirming that colonies from heterospecific pairings of both mating types readily occur, with an observed predominance in individual alates with ‘F1 Hybrid G’ genotypes. Such an observation contradicts the initial assumption of an asymmetrical introgression of *C. gestroi* genes within *C. formosanus* populations (= ‘F1 Hybrid F’ as the primary expected outcome), owing to a prezygotic behavioural bias by *C. gestroi* and *C. formosanus* males, in which both preferentially engage in tandem behaviour with *C. formosanus* females under laboratory conditions and in the field [30,52,53]. However, alate type collection may rely, in part, on the chance of established mature colonies in proximity to the monitored area. In addition, as ‘F1 Hybrid F’ colonies grew more slowly than ‘F1 Hybrid G’ in the laboratory, it is possible that such a delay in maturation may bias alate production in field colonies, with a resulting dominance in numbers of ‘F1 Hybrid G’ alates over ‘F1 Hybrid F’, instead of the expected opposite. Long-term monitoring over a wider geographic area will be necessary to elucidate this potential asymmetrical observation. Regardless, both locations confirmed F1 hybrid colony viability and the ability of both ‘F1 Hybrid F’ and ‘F1 Hybrid G’ colonies to reach maturity in the field and produce alates in various proportions, where postzygotic mechanisms may, in part, compensate for prezygotic behavioural biases, concerning the potential gene flow between the two species.

Field-collected F1 hybrid alates were predominantly female (9.9:1 ratio in Taiwan, 3.2:1 ratio in Florida), indicating limitations for F1 hybrid males. First, colonies may produce alates with an altered sex ratio compared with the ≈ 1:1 ratio observed in parental *Coptotermes* species ([51,60], current study), implying a putative altered nymphal development for hybrid individuals, as previously documented in hybrid workers [64] and in hybrid soldiers [58,65]. Second, individuals of both sexes could be produced in equal numbers, but poor male survivorship may effectively result in female-biased flights. The rapid death of F1 hybrid males in laboratory backcross attempts further supports this possibility. Third, as a corollary, weak F1 hybrid male alates may be relatively poor fliers, and therefore, their ability to reach the monitoring light trap may pale in comparison to F1 female alates. The rearing of laboratory F1 colonies to maturity would be required to investigate the causality of the observed effective biased sex ratios both in Taiwan and Florida. The low abundance/survival of F1 hybrid male alates implies that it is mostly F1 hybrid females that would have the opportunity to mate back with either one of the parental species in backcrossing events. In the current study, F1 hybrid alates were collected during many co-dispersal events with both or at least one of the parental species. Remarkably, the handful of advanced hybrid alates (> F1) collected in Taiwan confirmed that both maternal lineages were maintained within the hybrid zone and obtained preliminary evidence for the occurrence of the two F1 hybrid backcross possibilities with the two parental species. Thus, the combined observations in Taiwan and Florida confirmed the existence of introgressive hybridization between *C. formosanus* and *C. gestroi*, where F1 hybrid female alates from either mating combination have the opportunity to backcross with males of either parental species, i.e. all mating combinations for introgressive events remain possible, if given the opportunity.

While laboratory F2 colony viability has yet to be observed in Taiwan [60], Florida F2 hybrid laboratory colonies displayed some viability when established with an F1 hybrid female of either ‘F1 Hybrid F’ or ‘F1 Hybrid G’ origin. While all but one surviving F2 hybrid laboratory colony in Florida displayed the *C. gestroi* maternal lineage, it is unclear if this result stemmed from the initial alate sampling bias, as the majority of collected hybrid alates originated from a field-established ‘F1 Hybrid G’ colony (or colonies), or if F2 hybrid colonies established with ‘F1 Hybrid F’ females were not as viable as those established by ‘F1 Hybrid G’ females. Thus, despite apparent levels of genetic incompatibilities between the two *Coptotermes* species, which may be the cause of relatively poor F2 hybrid colony viability, the existence of advanced hybrid alates in the field in Taiwan and viable F2 hybrid laboratory colony in Florida both support the notion that compatible and viable hybrid mating combinations are likely to repeatedly occur through a numbers game of large dispersal flight events [50]: even if most hybrid pairings fail among the millions of attempts over time, it will eventually take just one successful mating to further allow the introgression process, if not already occurring. Ongoing introgression is therefore expected in areas where the geographical distributions of these *Coptotermes* species overlap, which has critically expanded in the past few decades in multiple locations around the world [44]. Furthermore, F1 hybrid colonies have a broad thermal tolerance that encompasses the minimal/maximum thermal tolerances of the two parental species [55,56]. This implies that hybrid colonies can establish within the cumulative potential distribution ranges of the two parental species, increasing the likelihood of establishment success as their spread is further facilitated by human-mediated activity [41,61].

Notably, both invasive species are readily dispersed by infesting privately owned leisure boats [66]. More than one million such boats are registered in the state of Florida [67], and south Florida is considered the ‘yachting capital of the world’, with more than 1 00 000 registered yachts and innumerable visiting maritime vessels with international origins visiting and docking annually. Such activity has resulted in the unintentional and, perhaps more importantly, uncontrolled introduction of countless invasive species in Florida, including *C. gestroi* and *C. formosanus* [40,68]. Furthermore, as thousands of private boats are docked and moored in canals and marinas within areas that are highly infested by one or both of these termite species in southeast Florida, it is unsurprising that hundreds of these vessels are routinely reported to be infested by either termite species annually [38]. The discovery of the first confirmed established F1 hybrid colony in Florida within close proximity to a boat marina area is most likely not random, as such locations represent primary gateways for invasive species to enter and disperse from. As hundreds of thousands of these boats are now within the overlapping distribution of both parental species in south Florida, where ongoing hybridization is likely to occur, it will be a matter of time for such introgressive hybridization to make its way outside of Florida, if it has not already. Functionally, the southeastern Florida metropolitan environment is not only in the process of becoming saturated by these two invasive species [45], but it is now also in the unique position to be a primary exporter of these two destructive structural pests, and now their hybrids, on a global scale.

While it is currently possible to identify hybrid termites morphologically and genetically for F1 hybrid alates and for F1 and F2 soldiers ([58,60], current study), morphological identification may become unreliable over several generations of backcrossing with either parental species, which may blur species delineation within the near future as a case of reticulate evolution. Currently, F1 alates can be morphologically differentiated from either parental species using the antennal spot as a reliable marker for live and preserved samples [60] and an intermediate abdominal colour hue for live specimens. Similarly, F1 and F2 soldiers can be morphologically differentiated from soldiers of the two parental species [58]. This information is key in facilitating the early detection of established hybridized *Coptotermes* population outside of their expected range where the two parental species overlap in distribution, as a result of their predicted dispersal via human-mediated activity. However, as observed in the current study, the fontanelle shape in hybrid soldiers beyond the F1 generation becomes an unreliable character for differentiation owing to its wide variability stemming from developmental instabilities [69]. An expansion of genetic markers will be necessary to trace back potential hybridization events for populations with an introgression history. This also implies that using mitochondrial genes alone for species identification is no longer a valid approach in these areas. From a pest control perspective, *C. formosanus* and *C. gestroi* structural infestation may be approached differently by pest management providers [70], and it is presently unknown if current pest management approaches will be similarly effective against hybrid colonies, as their destructive potential is equivalent to the two parental species [54]. Therefore, the societal impact of *Coptotermes* hybrids will most likely be compounded by the one already inflicted by the two destructive parental species.

To conclude, the ongoing human-mediated spread of *C. formosanus* and *C. gestroi* is not only irreversible but is also expected to further accelerate as invasive *Coptotermes* populations now serve as bridgeheads for further spread [35,36,40,41,44]. The incipient introgressive hybridization process described in the current study is an additional concern in the context of potential long-term evolutionary and ecological consequences, as gene flow between the two species at several locations around the world, including south Florida, has the potential to facilitate further expansions in the introduced range owing to the wide thermal tolerance of hybrid colonies [55,56]. As events of human-mediated hybridization between organisms continue to increase with global human activity [19,23,26], the *C. gestroi* × *C. formosanus* hybridization in Florida has the potential to be one with significant societal impacts [48]. Nevertheless, the *Coptotermes* hybrid system also represents novel genetic and symbiotic interactions [64,71] in a eusocial insect with high phenotypic plasticity [72], which could provide a unique evolutionary model to study.

## Data Availability

Most source data are provided with this paper within the main text and electronic supplementary material S1, including supplementary methods [73]. Additional data can be found on the Dryad Digital Repository [74].
